# A trait database for the characterization of diatom communities in disconnected pools

**DOI:** 10.1038/s41597-026-07836-6

**Published:** 2026-07-10

**Authors:** Guillermo Quevedo-Ortiz, Fernanda Gonzalez-Saldias, Julie Crabot, Frédéric Rimet, Joan Gomà, Núria Bonada

**Affiliations:** 1 FEHM-Lab (Freshwater Ecology, Hydrology and Management), Departament de Biologia Evolutiva, Ecologia i Ciències Ambientals, Facultat de Biologia, Universitat de Barcelona (UB), Diagonal 643, 08028 Barcelona, Spain; 2 Institut de Recerca de la Biodiversitat (IRBio), Universitat de Barcelona (UB), Diagonal 643, 08028 Barcelona, Spain; 3 SHE2 (Surface Hydrology, Erosion and Ecology), FEHM-Lab, Institute of Environmental Assessment and Water Research (IDAEA), CSIC, Jordi Girona, 18-26, 08034 Barcelona, Spain; 4 Department of Biodiversity and Environmental Management, Universidad de León, León, Spain; 5 UMR Carrtel, Université Savoie Mont Blanc, INRAE, France

## Abstract

Trait-based approaches are increasingly used in ecology to understand biodiversity responses to environmental changes. However, information on diatom traits, particularly in temporary aquatic systems, remains limited. Here, we present DIATPOOL, the first database focused on diatom traits from disconnected pools. DIATPOOL includes 17 morphological, ecological, and physiological traits, derived from literature and expert knowledge, including size, shape, apical constriction, dispersal capacity, ecological guilds, colony formation, or habitat tolerance. The database also includes an initial inventory of diatom species, their relative abundances, and environmental characteristics of disconnected pools sampled across the Iberian Peninsula. DIATPOOL provides an open and standardized resource to explore diatom biodiversity and functional traits in temporary and intermittent aquatic systems, offering a valuable framework for ecological, taxonomic, and bioassessment studies in these transitional habitats.

## Background & Summary

Over the last decades, trait-based studies have gained considerable attention in ecology due to their ability to predict biodiversity responses to environmental conditions^1–4^. Traits, whether used individually or in combination, allow for a straightforward characterization of community assembly under different environmental scenarios^5–7^. For example, they are useful in anticipating community shifts in response to global change drivers such as eutrophication or deforestation^8–10^. In general terms, traits can be defined as any heritable morphological, ecological, physiological, or phenological characteristic measurable at the individual level^11^. This definition encompasses a wide variety of characteristics related to species morphology, such as size, physiology, behavior (e.g., mobility), and life history traits (e.g., spore formation)^12–15^. The use of traits requires a detailed and careful preliminary characterization for each group of organisms^13,16^. Although several trait databases are already available and cover a wide range of organisms, including fish^17^, macroinvertebrates^4^, spiders^18^, and crustaceans^19^, there is still a need to create, complete, and expand trait information for many other organism groups.

Diatoms are photoautotrophic unicellular microalgae, solitary or colonial, that inhabit most aquatic and moist terrestrial environments^20,21^. They represent an extremely diverse group, with estimates ranging between 100.000 and 200.000 species^22,23^, of which only about 64.000 have been formally described^24,25^. Currently, several trait databases classify diatoms according to life-forms, size classes, ecological guilds, or indicator values, among others^14,26,27^. Their effectiveness as predictors of environmental conditions has been widely demonstrated across studies^28–30^. For example, it is known that small, mobile, and low-profile species are more abundant during the summer in alpine rivers^31^. It has also been observed that adhesion strategies appear to confer a greater competitive advantage under more unstable environmental conditions, compared to diatoms of lower adhesion capacity (e.g., motile and planktonic)^32^. Additionally, small diatom species tend to disperse more successfully over long distances, primarily due to wind transport^33,34^. Furthermore, mobile taxa with weak substrate attachment can disperse farther than those that are strongly attached or planktonic^35^. However, collecting information on traits for small and diverse organisms such as diatoms is a highly complex task. The lack of data for rare or infrequent taxa, the existence of species complexes, and information based on researchers’ expertise pose significant challenges for characterizing these traits^14,36^. Moreover, given that diatoms are good bioindicators, it is crucial to compile these traits, as they can offer valuable insights into the use of diatoms in biomonitoring^14,27,37^.

Temporary rivers, also known as Intermittent Rivers and Ephemeral Streams (IRES), are highly dynamic fluvial ecosystems that can harbor a rich diversity of diatoms^38^. These systems are characterized by alternating wet and dry phases^39,40^. While in the wet phase water flows between different sections of the river, the dry phase is characterized by a complete loss of flow. During the latter, it is common to find disconnected pools, which may either reconnect or dry out depending on environmental conditions^41^. Pools therefore constitute transitional habitats between aquatic and terrestrial environments, with a strong capacity to host unique diatom communities^38,40,42,43^. Despite this, no database currently exists that specifically addresses the diversity of diatom traits in disconnected temporary pools. This is especially important considering that temporary rivers are more prevalent globally than perennial rivers^44^, and that flow intermittence is projected to increase^45^, leading to a greater occurrence of disconnected pools. To address this gap, we present DIATPOOL, a database focused on the traits of diatoms from disconnected pools of temporary rivers. While information on certain traits (e.g., guilds, indicator values, and coloniality) can be found online, other relevant data remains scattered, incomplete, or unpublished. In DIATPOOL, we have compiled, updated, and organized traits related to the potential adaptation of diatoms to the environmental conditions of disconnected pools. Additionally, we propose new traits that may be useful for characterizing such habitats. Our goal is therefore to provide a unique resource that facilitates the integration of traits related to flow intermittence in ecological research and highlights the importance of these transitional habitats for diatom diversity.

## Methods

### Diatom species in DIATPOOL

**Fig. 1:**
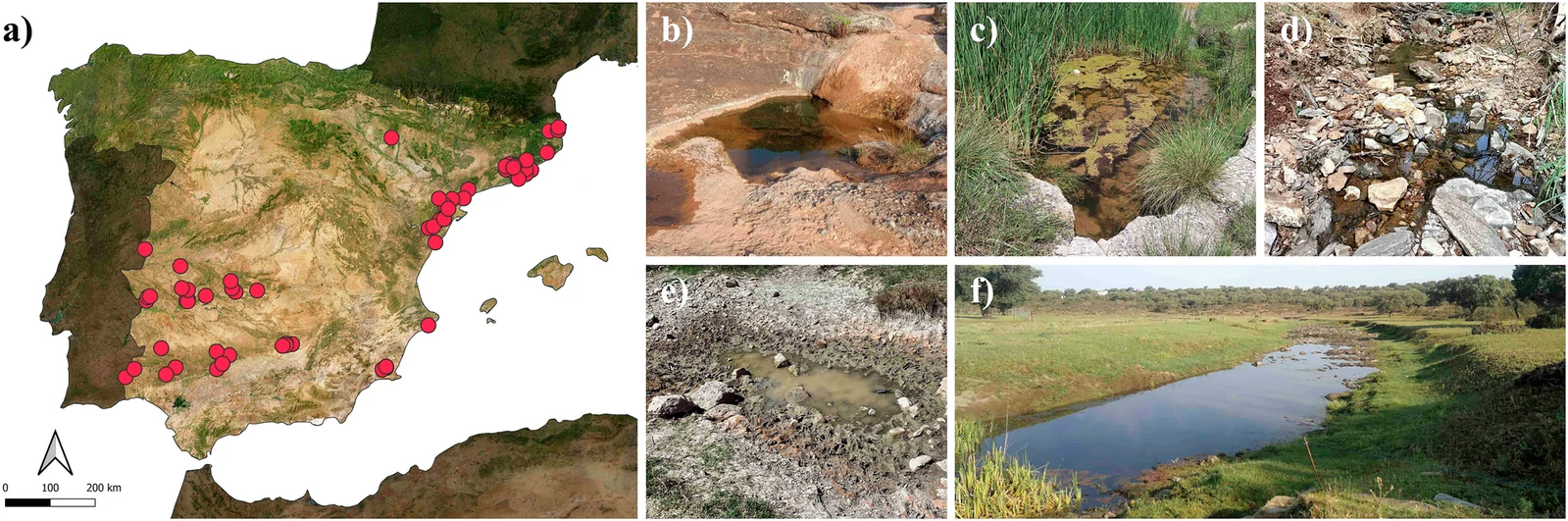
Diversity of disconnected pools sampled. (**a**) Map of the Iberian Peninsula with the locations of the pools (red circles); (**b**) Riera de Llogari (Catalonia); (**c**) Río Bergantes (Valencia); (**d**) Arroyo de Valdeazores (Andalusia); (**e**) Rambla del Garruchal (Murcia); (**f**) Río Gibranzos (Extremadura).

The DIATPOOL database is associated with an initial list of diatom species characteristic of disconnected pools (see the taxonomy section in the DIATPOOL matrix, available in Data Records), which is expected to grow as more pools are sampled. This inventory of diatoms was obtained from successive samplings of benthic diatoms conducted in the Mediterranean region of the Iberian Peninsula (Fig. 1). Between May and July 2023 and 2024, a total of 66 disconnected pools with diverse characteristics (size, geology, degree of anthropogenic influence, climate, etc.) were sampled (Metadata_pool, available in Data Records). All pools were in an arheic state, i.e., completely disconnected, without flow but with surface water^39,46^. In cases where there was more than one (within 200 m), the most representative one was always selected, recording the number of other pools and the distance to the nearest one. Samples were collected by scraping seven submerged stones (approx. 15 cm² each) with a toothbrush and subsequently preserved in 96% ethanol. In addition, physicochemical variables were recorded *in situ* in each pool using a multiparameter probe (YSI Professional Plus Multiparameter Probe), and a water sample was collected for nutrient analysis in the laboratory (Metadata matrix, available in Data Records).

All diatom samples were treated in a 33% hydrogen peroxide bath at 90 °C. Drops of hydrochloric acid were added to remove potential calcium carbonate inclusions. The material was left to settle for 48 hours, followed by successive rinses with distilled water, removing the supernatant each time. A portion of the resulting material was placed onto glass slides and allowed to dry for 24 hours. The dry slides were then mounted with Naphrax© resin. Samples were examined under a Zeiss Polyvar microscope at 1000 × magnification using differential interference contrast and a digital camera. All samples were taxonomically identified to the species level using online publications and the main diatom monographs^47–52^. The taxonomic list consisted of a total of 385 species, including varieties. Additionally, a total of 400 valves were counted for each sample, and the relative abundance was calculated (Abundance Matrix, available in Data Records). Once the matrix was obtained, each species was assigned the traits described below, based on information from published sources, our own data, image interpretation, and expert evaluation.

### Traits in DIATPOOL

**Fig. 2:**
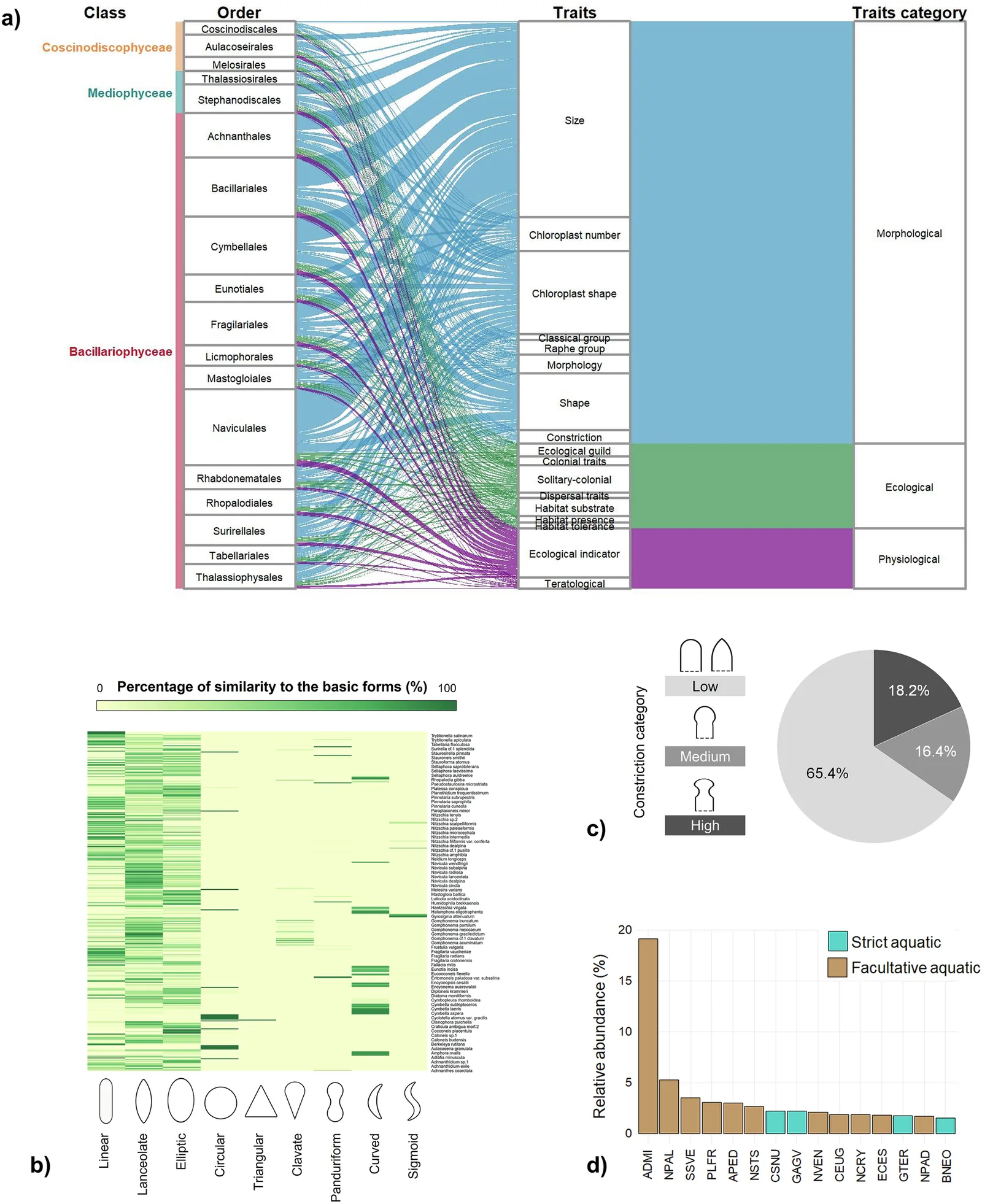
Diatom traits from disconnected pools are considered in DIATPOOL. (**a**) Sankey diagram illustrating the proportional contributions of the different orders to the 17 traits described here, grouped into three main categories: morphological (light blue), ecological (green), and physiological (light violet). (**b**) Main diatom shapes (linear, lanceolate, elliptic, circular, triangular, clavate, panduriform, curved, sigmoid) and their representation in each species. Original drawings by the authors were inspired by Lange-Bertalot and colleagues^73^. Diatoms with a single, well-defined shape corresponding to one of the described morphotypes are shown in dark green (e.g., 100% circular), whereas taxa exhibiting shape variability are represented by lighter shades, reflecting the proportion of each morphotype contributing to their overall form (e.g., 60% lanceolate, 40% clavate). (**c**) Classification of diatoms according to the degree of apical constriction: low (light gray), medium (gray), and high (dark gray). (**d**) The 15 most abundant diatom species, representing their overall relative abundance (%) and their tolerance to aquatic environments. Diatoms that are strictly aquatic are shown in blue, while facultative aquatic diatoms are shown in brown. ADMI (*Achnanthidium minutissimum*), NPAL (*Nitzschia palea*), SSVE (*Staurosira venter*), PLFR (*Planothidium frequentissimum*), APED (*Amphora pediculus*), NSTS (*Nitzschia soratensis*), CSNU (*Craticula subminuscula*), GAGV (*Gomphonema angustivalva*), NVEN (*Navicula veneta*), CEUG (*Cocconeis euglypta*), NCRY (*Navicula cryptocephala*), ECES (*Encyonopsis cesatii*), GTER (*Gomphonema tergestinum*), NPAD (*Nitzschia palea* var. *debilis*) and BNEO (*Brachysira neoexilis*).

The DIATPOOL database includes 17 traits (8 morphological, 7 ecological, and 2 physiological), with variable trait categories derived from previously published trait databases and complemented by original traits specifically selected for their relevance to disconnected pools. To facilitate the understanding of the data structure, Fig. 2a (Sankey diagram) summarizes the contributions of the different diatom orders sampled across the three main categories, linked to the 17 traits defined in DIATPOOL.

Below, we describe the main traits selected for the construction of DIATPOOL. These traits are expected to reflect adaptations to the hydrological, physicochemical, and geomorphological conditions characteristic of disconnected pools^41^. Some of them have been used to characterize lentic ecosystems, such as wetlands, ponds or lakes, whereas others are typically used in lotic ecosystems^53^. Although other traits, such as physiological^27^, transcriptomic, or metabolomic ones^54^, could provide valuable insights into the adaptations of diatoms to these environments, they were not included in the current version of the database due to the limited availability of such information. Nevertheless, DIATPOOL can serve as a solid foundation that may be expanded in the future as more data on these and other types of traits become available.

#### Size [morphological]

This trait classifies diatom species according to cell size. Given the high dynamism of temporary rivers, a higher presence of larger species is likely to be observed in disconnected pools due to their lentic conditions. Cell length, width, and thickness (µm) values were obtained from published literature^14,42,48–51,55–86^. For this reason, we propose five alternative morphological forms.

- *Length and width (µm):* Corresponds to the average length and width of the valve, calculated as (minimum value + maximum value)/2. Most reference values for each diatom species considered in DIATPOOL (see below) were obtained from previously compiled data^14,87^, primarily based on Krammer and Lange-Bertalot^48–51^. We also updated some values and added others for new species. (Continuous variable).
- *Logarithmic length and width (log):* Corresponds to the base 10 logarithm of the average length and width of the valve, calculated as log10 (average value + 1). This calculation normalizes the data and reduces the influence of extreme values, facilitating statistical comparisons and multivariate analyses. (Continuous variable).
- *Length and width (µm intervals):* Refers to the size interval to which each measurement belongs. Length intervals were set at 5 µm (e.g., [0,5], [5,10], [10,15], etc.), whereas width intervals were set at 1 µm (e.g., [0,1], [1,2], [2,3], etc.). These intervals reflect intraspecific variability and are useful for representing the morphological plasticity of each species. (Categorical variable).
- *Length and width (group and category):* Classification of length and width measurements into different groups. Based on our database, six types were defined according to size. (Categorical variable).
- *Length (group, category, intervals):* 1 Very small [0, 10); 2 Small [10, 25); 3 Medium [25, 50); 4 Large [50, 100); 5 Very large [100, 200); 6 Giant [200, > 200).
- *Width (group, category, intervals):* 1 Very small [0, 5); 2 Small [5, 10); 3 Medium [10, 20); 4 Large [20, 30); 5 Very large [30, 50); 6 Giant [50, > 50).
- *Size (group and category):* Classification of diatom size based on the length and width groupings. From the previously calculated categorical values (1 Very small – 6 Giant), 11 easily identifiable new categories were established. This involves averaging the two groups to obtain an intermediate general size value (length group + width group)/2). This method is useful for characterizing simply and understandably species with uneven measurements. Other studies have used biovolumes to classify diatom size^88^, which allows for more precise estimates. However, thickness information is often unavailable and requires expert calculation and various formulas^14^. Our formula aims for an intuitive and practical grouping of diatom sizes. For instance, diatoms with identical length and width groups maintain that category (e.g., length 2 Small + width 2 Small → size 2 Small; example: *Achnanthidium exile*). Diatoms with slight differences between the two measurements obtain an intermediate value (e.g., length 3 Medium + width 2 Small → size 2.5 Medium/Small; example: *Brachysira brebissonii*). Diatoms with notable differences receive a middle category (e.g., length 3 Large + width 1 Very small → size 2 Medium; example: *Nitzschia acicularis*). Finally, species with extremely unequal measurements receive an approximate intermediate category (e.g., length 6 Giant + width 2 Small → size 4 Large; example: *Ulnaria ulna*). (Categorical variable).
- *Size (group category):* 1 Very small; 1.5 Very small/Small; 2 Small; 2.5 Small/Medium; 3 Medium; 3.5 Medium/Large; 4 Large; 4.5 Large/Very large; 5 Very large; 5.5 Very large/Giant; 6 Giant.
- *Thickness (µm):* This parameter refers to the thickness of the frustule. Since it is not usually reported in floras, the values were obtained from precise measurements and/or estimates provided by Rimet and Nicolosi Gelis^14,87^. (Categorical variable).

#### Chloroplast number [Morphological]

This trait classifies diatoms according to the morphological characteristics of their chloroplasts. Since these organelles provide useful information about different lineages^53^, their number may vary depending on environmental conditions (e.g., light) in disconnected pools. These characterizations were obtained from published literature^82,89–93^ and the Diat.barcode v15.2 database^87^. However, chloroplast studies still require further characterization for many species (Categorical binary variables, not mutually exclusive):

- *Chloroplast number*: 1 chloroplast; 2 chloroplasts; 3 chloroplasts; 4 chloroplasts; 5 chloroplasts; 6 chloroplasts; 7 chloroplasts; more than 7 chloroplasts; Variable (unspecified) chloroplasts. The last category includes species with a defined number of chloroplasts (e.g., 1 or 2), but for which higher counts have also been reported without specifying the exact number.

#### Chloroplast shape [Morphological]

Similarly, the shape of chloroplasts can also provide important information about diatom characteristics. This trait remains largely unexplored both in disconnected pools and in other environments, and therefore future studies will help confirm its potential. Based on the different shapes observed by Diat.barcode v15.2^87^ and from published literature^82,89–93^, we defined a set of common forms. However, it should also be noted that further chloroplast studies are still needed:

- Plate-like; Strip; Ribbon; Discoid; H-shaped; C-shaped; Lobed; Spathulate; Granular; Constricted_centre; Elongate/rod-like; Stellate; Oval; Butterfly-shaped; X-shaped; Cup-shaped; Paired_plates; Pair_lobes; Much_lobes; Picture_TCC534; Picture_TCC483.

#### Classical group [morphological]

This trait corresponds to the traditional classification of diatoms into centric and pennate forms^83,94–96^. Although this division is not phylogenetic (e.g., centric diatoms do not form a monophyletic group), it provides valuable information on morphology and ecology. Considering that disconnected pools originate from temporary rivers (lotic systems) but function as small ponds or lakes (lentic systems), this trait can be highly informative (particularly in multi-habitat samples, e.g., benthic, planktonic, or periphytic samples). (Categorical binary variables, mutually exclusive).

- *Centric*: Species with radial symmetry, typically circular, oval, triradiate, triangular, quadrate, or polygonal in shape (rarely elongated). They are usually associated with lentic environments and exhibit a primarily planktonic lifestyle. Sexual reproduction is morphologically oogamous in most genera^53^.
- *Pennate*: Species that display bilateral symmetry around a sternum, most often elongated in shape. They are generally associated with benthic habitats and may or may not have the ability to move through a raphe (a slit-like structure). Sexual reproduction is morphologically isogamous in most genera^53^.

#### Raphe group [morphological]

This trait classifies diatom species according to the presence and type of raphe, a structure that enables cell motility^89^. Since diatoms have been shown to actively move toward areas of higher moisture^97^, this trait may represent an adaptive advantage in disconnected pools, where partial drying is frequent. Instead, centrals could dominate larger disconnected pools, as they are usually found in lentic waters such as lakes or ponds^98,99^. We defined five main categories according to the published literature^83,96,100–104^. (Categorical binary variables, mutually exclusive).

- *Centric:* Radially symmetric species that completely lack a raphe. They lack significant motility. Although centric diatoms, like araphid species, do not possess a raphe, their circular morphology is highly distinctive in planktonic environments (e.g., *Aulacoseira*, *Cyclotella*).
- *Araphid*: Species that completely lack a raphe. They lack significant motility (e.g., *Fragilaria*, *Diatoma*).
- *Monoraphid*: Species in which only one of the two valves bears a raphe (e.g., *Achnanthidium*, *Cocconeis*). Motility is limited and slow, and cells are typically attached to the substrate most of the time via mucilage.
- *Biraphid:* Species in which both valves possess a raphe. They usually exhibit good motility (e.g., *Tryblionella*, *Cymbella*).
- *Brachyraphid*: Some authors distinguish certain genera from the brachyraphid diatoms, which appear to represent an early branch within the raphe-bearing diatom lineage^23,105^. In these cases, species have a reduced or rudimentary raphe, usually located at the ends of the two valves. Consequently, motility is generally limited (e.g., *Eunotia*, *Peronia*).

#### Morphology [morphological]

This trait classifies diatom species according to morphological similarity, following the grouping proposed by Diatoms.org^83^. These groups are not strictly related phylogenetically, but they provide a simple means of grouping based on morphological traits, raphe presence, symmetry, and other features^83,96,100–104^. This approach can be particularly useful in disconnected pools, where environmental conditions may confer an advantage to certain related groups. Species were classified into nine categories. (Categorical binary variables, mutually exclusive).

- *Araphid:* Species with bilaterally symmetrical valves, both lacking a raphe. They lack significant motility (e.g., *Meridion*, *Ulnaria*).
- *Asymmetric biraphid:* Species with valves asymmetric along the apical axis, the transapical axis, or both. Both valves bear a well-developed raphe (e.g., *Cymbella*, *Gomphonema*).
- *Centric:* Species with radially symmetrical valves (symmetry around a point), both lacking a raphe. They lack significant motility (e.g., *Melosira*, *Discostella*).
- *Epithemioid:* Species with bilaterally symmetrical valves, but asymmetric along the apical axis. Both valves bear a raphe enclosed in a canal, usually near the margin (e.g., *Epithemia*, *Rhopalodia*).
- *Eunotioid:* Species with bilaterally symmetrical valves, often asymmetric along the apical axis. Both valves bear a short raphe, located on the mantle and the valve face. They usually exhibit weak motility (e.g., *Eunotia*).
- *Monoraphid:* Species with bilaterally symmetrical valves, only one of which bears a raphe (e.g., *Achnanthidium*, *Planothidium*).
- *Nitzschioid:* Species with bilaterally symmetrical valves, typically symmetrical along both the apical and transapical axes. Both valves have a raphe enclosed in a canal, usually near the margin (e.g., *Hantzschia*, *Nitzschia*).
- *Surirelloid:* Species with bilaterally symmetrical valves, both bearing a raphe enclosed in a canal, often elevated on a keel (e.g., *Entomoneis*, *Surirella*).
- *Symmetric biraphid:* Species with bilaterally symmetrical valves, symmetrical along both the apical and transapical axes. Both valves bear a well-developed raphe. They usually exhibit high motility (e.g., *Adlafia*, *Hippodonta*).

#### Shape [morphological]

This trait considers the overall shape of diatom valves. It is a non-phylogenetic trait aimed at identifying morphological similarities that may reflect convergent adaptations to the specific conditions of disconnected pools. For example, some shapes may be advantageous for moving through sediments, while others, such as curved or hook-shaped forms, may facilitate passive dispersal between pools^38,97^. The classification was established based on the main forms described in the original species descriptions and referenced in various available monographs^42,52,56,59,73,77,80,106^. Although the rhomboid shape was used in several guides, it was not included because it is often similar to the lanceolate shape. Each species was assigned a percentage of membership to its main shape, ensuring the total sum reached 100%. This means that diatoms with basic forms are composed of 100% of a single form (e.g., 100% circular), whereas others are composed of a combination of basic forms (e.g., 70% curved and 30% lanceolate). Figure 2b represents the distribution and pattern of each shape across the different diatom species found in our samples. This trait includes nine categories. (Percentage variable).

- *Linear:* A rectangle with a semicircle at each end (e.g., *Fragilaria tenera*, *Ulnaria capitata*).
- *Lanceolate:* An elliptical shape tapering at the ends, like a spear (e.g., *Achnanthidium exile*, *Brachysira aponina*).
- *Elliptic:* A simple oval or elliptical shape (e.g., *Cocconeis euglypta*).
- *Circular:* A perfectly round shape (e.g., *Discostella pseudostelligera*, *Stephanodiscus medius*).
- *Triangular:* An equilateral triangle (e.g., *Cyclotella alvarniensis*).
- *Clavate:* A club- or cone-shaped form, wide at the top and narrow at the base (e.g., *Gomphonema truncatum*, *Gomphonema capitatum*).
- *Panduriform:* An hourglass-like shape, narrow in the center and wider at top and bottom (e.g., *Surirella librile*).
- *Curved:* A curved, arched, or crescent-shaped form (e.g., *Cymbella affinis*, *Amphora ovalis*).
- *Sigmoid*: A smooth “S”-shaped curve (e.g., *Nitzschia sigmoidea*).

#### Constriction [morphological]

This trait classifies diatom species based on the degree of apical constriction. Similar to the shape trait, it is not a phylogenetically-related trait but a functional criterion aimed at detecting potential convergent adaptations to highly dynamic environments such as disconnected pools. Apical constriction may influence both attachment and the ability of cells to move among sediment microparticles in search of more humid microhabitats. To code this trait, the apical poles of all identified species were analyzed using original species descriptions and the main available monographs^42,52,56,59,73,77,80,106^. Figure 2c shows a diagram of the constriction degree and their representation in diatoms from some disconnected pools. As this is a qualitative trait, further morphometric studies could refine and expand this information in future research^107^. (Categorical binary variable, mutually exclusive). Based on the estimated constriction degree, three types were defined.

- *Low:* Diatoms with very low or no apical constriction (e.g., *Tryblionella levidensis*, *Sellaphora nigri*).
- *Medium:* Diatoms with medium apical constriction (e.g., *Navicula subalpina*, *Gomphonema micropus*).
- *High:* Diatoms with high apical constriction (e.g., *Gogorevia exilis*, *Pinnularia saprophila*).

#### Ecological guilds [ecological]

This trait characterizes ecological guilds, which are groups of taxa that share similar traits and exploit similar resources^108^. Given the high diversity of habitats in disconnected pools (water column, sediments, macrophytes, rock surfaces, etc.), it is likely that different species share distinctive traits that allow them to cope with the various environmental challenges. We adopted the guilds proposed by Rimet and Nicolosi Gelis^14,87^, modified from Passy^26^, due to their robustness and wide use in ecological studies^14,26,42,47–51,62,71,72,82,89,109–152^. (Categorical binary variables, mutually exclusive).

- *High profile*: This guild includes tall species such as erect, filamentous, branched, chain-forming, tube-forming, stalked taxa, and colonial centrics. These species are typically adapted to high nutrient concentrations and low flow velocities.
- *Low profile*: This guild includes short-stature species such as prostrate forms (adhering to the substrate with the entire valve surface), adnate forms (apically attached but parallel to the substrate), erect forms (apically attached but perpendicular to the substrate), and slow-moving species. This group is generally well adapted to high flow velocities and low nutrient concentrations.
- *Motile***:** This guild is composed of fast-moving species, mainly from the genera *Navicula*, *Nitzschia*, *Sellaphora*, and *Surirella*, as well as additional taxa from the orders Mastogloiales, Naviculales, Rhopalodiales, and Surirellales.
- *Planktonic*: This guild includes taxa typically found in lentic environments, characterized by morphological adaptations that prevent sedimentation.

#### If colonial [ecological]

The trait refers to the capacity of certain diatoms to form colonial aggregations^14,21,89^. Given that disconnected pools function as small lentic systems, colony formation may represent an advantageous strategy^14,26,42,47–51,62,71,72,82,89,109–152^. For example, it is known that a larger colonial size can be an adaptation that slows down sedimentation in different environments^153,154^. (Categorical binary variables, not mutually exclusive).

- *Colonial*: This category includes species capable of forming colonies. This includes representatives of several genera, such as *Melosira*, *Eunotia*, and *Fragilaria*.
- *Non-colonial*. This category includes species that do not form colonies. This includes representatives of several genera, such as *Mastogloia*, *Achanthes*, and *Surirella*.
- *Both:* This category includes species that can occur either as solitary cells or in colonial arrangements depending on environmental conditions or life cycle stage.

#### Solitary cells (anchored) and colonial type [ecological]

Solitary and colonial diatoms exhibit different life-forms depending on their mode of attachment (in solitary cells) or the structure of the colony^14,26,42,47–51,62,71,72,82,89,110–152,155^. Some species may alternate between forms throughout their life cycle^14^. The differentiation between these two lifestyles may be related to various environmental aspects of disconnected pools. For example, an increase in the number of solitary cells may be observed in pools nearing desiccation (greater motility), or even in colonial forms (development of protective mucilage). We classified these lifestyles into the following categories, following Rimet and Bouchez^14^, and based on Round and collaborators^89^.

- *Solitary cells (attached):* This category includes diatoms that attach to the substrate through one or several mechanisms. (Categorical binary variables, not mutually exclusive).
- *Adnate:* Firmly attached by the valve face or girdle view.
- *Pedunculate:* Produce a stalk or pad attached to the substrate.
- *Pad:* Produce mucilage at one pole for attachment to the substrate.
- *Stalk:* Use apical pore fields to produce a stalk for attachment.
- *Colonial type:* This category includes the diversity of colony arrangements found in diatoms. (Categorical binary variables, not mutually exclusive).
- *Ribbon:* Pennate cells juxtaposed by their valves and connected by spines or mucilage secretions along the valve face.
- *Zig-zag:* Pennate cells connected by mucilage at opposite poles.
- Rosette: Pennate cells produce a short stalk at one pole, forming fan- or rosette-like colonies.
- *Arbuscular:* Stalks produced at one or both poles that branch to form arbuscular colonies.
- Mucous tubule. Colonies develop within mucilaginous tubes, with rows of cells moving internally.
- *Chain colonies:* Centric cells juxtaposed by their valves, connected by spines, granules, or polysaccharide filaments.
- *Stellate:* Pennate cells connected by mucilage at adjacent poles, forming a star-like arrangement.
- *Others:* Other types of colonies.

#### Dispersal capacity [ecological]

This trait considers the ability of diatoms to disperse among optimal habitats. It is related both to the speed of colonization following a rewetting event and to high dispersal rates^14,38,43,59,82,156^. This capacity is key for the spatial and temporal colonization of disconnected pools^38^, being reflected in different resistance and resilience strategies. We adapted the “mobile” and “pioneer” traits from Rimet and Bouchez and Nicolosi Gelis^14,87^, incorporating new evidence on potential species. (Categorical binary variables, not mutually exclusive).

- *Motile:* This category includes diatoms capable of active movement via a raphe. This trait is particularly relevant in disconnected pools, as some diatoms can migrate to wetter microhabitats (e.g., hyporheic zone) in response to changes in water flow^157^.
- *Pool pioneer:* This category includes small pioneer species, originally defined in rivers (the *Achnanthidium minutissimum* complex and some *Amphora species*)^14,87^. Since they also display highly proliferative populations in pools, we retained these species along with additional ones. Specifically, we added other predominantly small- to medium-sized species with high proliferative capacity in disconnected pools, detected in disconnected pools (e.g., *Nitzschia palea*, *Mayamaea atomus*)^38,43,156^.

#### Habitat substrate [ecological]

This trait classifies diatom species according to the types of substrates they typically occupy^82^. Since disconnected pools can present a wide variety of microhabitats^39,46^, we defined a set of standard categories based on the type of sampling performed. This classification is based on and adapted from the results previously presented by Nicolosi Gelis^87^, focusing exclusively on substrate types. (Categorical binary variables, not mutually exclusive).

- *Benthic:* Species inhabiting bottom substrates, generally associated with sediments, stones, or submerged surfaces in aquatic environments. Our species list was collected from this substrate.
- *Planktonic:* Species predominantly found suspended in the water column.
- *Epipsammic:* Species living attached to sand grains, typically occurring in shallow or unstable sediment environments.
- *Epipelic:* Species growing on fine sediments (mud or silt).
- *Wet soil / Bryophytes / Intertidal zone:* Species associated with wet soils, mosses, or intermittently moistened substrates.

#### Habitat presence [ecological]

This trait classifies the main habitats where different diatom species can be found. Since disconnected pools act as transitional habitats between aquatic and terrestrial environments, it is reasonable to expect that diatoms typical of both habitats may be present^14,38,42,52,56–69,73,75–81,84–86,124,158–174^. To establish this classification, species lists from various sources were reviewed, recording the recurrent species. (Categorical binary variables, not mutually exclusive).

- *Aquatic:* Species identified in freshwater aquatic environments, regardless of flow intermittence. All species in this database were included in this category, as they were observed in aquatic habitats (i.e., disconnected pools).
- *Terrestrial/soil:* Species recorded in terrestrial or soil environments. Compared to aquatic habitats, the literature on this group is more limited^163,174^.
- *Aerial:* Species identified in aerial environments, either euaerial (on elevated rocks receiving moisture exclusively from the atmosphere) or pseudoaerial (on rocks moistened under conditions such as waterfalls or springs). These habitats remain poorly explored (compared to the others) and require further community characterization^66,110,175^.

#### Habitat tolerance [ecological]

This trait classifies diatom species according to their ability to inhabit exclusively aquatic environments^14,42,52,56–69,73,75–81,84–86,124,158–174^. Since disconnected pools may undergo processes of water flow reconnection and drying, facultative or strictly aquatic diatoms are likely to modify their frequency and abundance. In this way, we classify two main categories (categorical variables, mutually exclusive). Figure 2d presents the habitat tolerance (strictly aquatic or facultative) of the most abundant species included in our database. (Categorical binary variables, mutually exclusive).

- *Strict aquatic:* Diatoms recorded exclusively in freshwater species lists.
- *Facultative aquatic:* Diatoms commonly found in both freshwater species lists and non-aquatic environments, including terrestrial/soil, euaerial, or pseudoaerial habitats.

#### Ecological indicator values [physiological]

This includes ecological traits that inform about the environmental conditions in which diatom species occur^27^. These traits are widely used to assess water quality, ecosystem health, and other environmental aspects^27,47,56,71–73,89,111,114,120–123,125,126,130,131,134,139,150,176–277^. Incorporating this information into our database allows insights into species presence, absence, abundance, community composition, tolerance ranges, and their potential relationships with teratological forms. This trait could be very useful in disconnected pools, as these habitats may present environmental conditions very different from those of their source rivers (e.g., ions, temperature, nutrients, etc.). We adopted the classification proposed by Mertens and collaborators^27^. In addition, given their importance^278^, we provide a new metric related to the temperature tolerance of the diatoms found in disconnected pools. These values were calculated based on the maximal relative abundance observed in each species across the set of disconnected pools and may be refined in future studies. (Categorical variables):

- *pH (1–5, X):* 1 = Acidobiontic (optimal occurrence at pH < 5.5); 2 Acidophilous (mainly pH < 7); 3 Circumneutral (mainly around pH 7); 4 Alkaliphilous (mainly pH > 7); 5 Alkalibiontic (exclusively pH > 7); X Indifferent (no apparent optimum).
- *Salinity (1–7):* 1 Fresh (Cl- < 100 mg l^−1^, salinity < 0.2%); 2 Fresh-brackish (Cl- < 500 mg l^−1^, salinity < 0.9%); 3 Brackish-fresh (Cl- 500–1000 mg l^−1^, salinity 0.9–1.8%); 4 Brackish-fresh (Cl- 1000–5000 mg l^−1^, salinity 1.8–9%); 5 Brackish-marine (Cl- 5000–10000 mg l^−1^, salinity 9–18%); 6 Marine-brackish (Cl- 10000–17000 mg l^−1^, salinity 18–30%); 7 Marine (Cl- > 17000 mg l^−1^, salinity > 30%).
- *Nitrogen uptake metabolism (1–4):* 1 Nitrogen-autotrophic, tolerating very low concentrations of organically bound nitrogen; 2 Nitrogen-autotrophic, tolerating elevated concentrations of organically bound nitrogen; 3 Facultatively nitrogen-heterotrophic, needing periodically elevated concentrations of organically bound nitrogen; 4 Obligately nitrogen-heterotrophic, needing continuously elevated concentrations of organically bound nitrogen.
- *Oxygen requirements (1–5):* 1 Continuously high (~100% saturation); 2 Fairly high (>75% saturation); 3 Moderate (>50% saturation); 4 Low (>30% saturation); 5 Very low (~10% saturation).
- *Saprobity (1–5, X):* 1 Oligosaprobous; 2 β-mesosaprobous; 3 α-mesosaprobous; 4 α-meso-/polysaprobous; 5 Polysaprobous; X = Indifferent.
- *Trophic state (1–6, X):* 1 Oligotraphentic; 2 Oligo-mesotraphentic; 3 Mesotraphentic; 4 Meso-eutraphentic; 5 Eutraphentic; 6 Hypereutraphentic; X Indifferent.
- *Moisture preference (1–5):* 1 Rarely occurring outside water bodies; 2 Mainly in water, sometimes in wet places; 3 Mainly in water, also regularly in wet and moist places; 4 Mainly on wet and moist or temporarily dry places; 5 Nearly exclusively outside water bodies.
- *Temperature (1-5):* 1 The temperature at which the maximum relative abundance occurs in the disconnected pools. 1 Optimum at 0–10 °C; 2 Optimum at > 10–20 °C; 3 Optimum at > 20–30 °C; 4 Optimum at > 30–40 °C; 5 Optimum at > 40–50 °C.

Note: Additionally, the appendices of DIATPOOL include information related to temperature values (°C). Specifically, minimum, maximum, range, and mean temperatures are provided, as well as the temperature of the pool in which each species reached its maximum population abundance. We recommend continuing efforts to refine the calculation of diatom tolerance ranges, given that these remain unknown for the majority of species^279–281^.

#### Teratological form [physiological]

This trait includes diatom taxa prone to exhibiting teratological forms, which are morphological anomalies reflecting sublethal responses to environmental stress. These deformities are easily detected during routine taxonomic analyses^282^ and are frequently reported in the literature^14,80,232,283–372^. In disconnected pools, these bioindicators could provide valuable information, as these habitats often experience strong fluctuations in nutrient levels, pH, temperature, and oxygen, among other factors^41,373^. Based mainly on Falasco and collaborators, we included the occurrence of deformities to assess potential adaptive strategies of species under such dynamic environmental conditions^152^. Information was also compiled from specialized online resources on teratologies (e.g., La Tératothèque from IRAE^372^) through detailed analysis of diatom images. (Categorical binary variables, mutually exclusive).

- *Reported:* Taxa for which teratological forms have been documented in the literature and online resources.
- *Not reported:* Taxa for which teratological forms have not yet been documented.

Note: Complementarily, the appendices of DIATPOOL include information related to teratology. Specifically, the diversity of teratological forms (e.g., deformed valve outline or changes in striation patterns, costae, and septae), and their main causes (such as acid mine drainage or artificial growth conditions).

## Data Records

The DIATPOOL database, diatom abundances (Abundance_diatoms), and metadata (Metadata_pool) are available for use and download at Zenodo^106^: https://doi.org/10.5281/zenodo.20579411.

### DIATPOOL.xlsx

This file contains the main dataset. The first sheet includes an initial database of 385 diatom species identified in the disconnected pools. The first ten columns provide taxonomic characterization information, including OMNIDIA codes. In cases where some species lack code, alternative available codes (not used in OMNIDIA) have been assigned. These codes can be easily identified by filtering the Reference_CODE1 column in REFERENCE_TAXONOMY. The subsequent columns include the different biological, ecological and physiological traits previously described: size, classical group, raphe group, morphology group, shape, constriction, ecological guilds, cell type (solitary or colonial), dispersal capacity, habitat presence, habitat tolerance, ecological indicator and teratological form. Each trait category is highlighted with a distinctive color to facilitate identification and interpretation. Finally, the last columns include the bibliographic sources used for each trait, complementary data on the diversity and causes of teratology, as well as the taxonomic synonymy considered for certain species. Additional sheets provide simplified summaries of each section to facilitate understanding.

### Abundance_diatoms.xlsx

This file contains the matrix of relative abundances associated with the 385 species included in DIATPOOL. Percentages were calculated from counts of 400 valves per sample, corresponding to the analyzed pools.

### Metadata_pools.xlsx

This file provides the environmental characterization of the 66 disconnected pools used as reference sites for DIATPOOL. It includes different information:

- *Geographical*: autonomous community (Catalonia, Aragon, Valencia, Murcia, Andalusia, Castilla La Mancha and Extremadura), basin (Ebro, Internal basins Catalonia, Jucar, Segura, Guadalquivir, Guadiana and Tajo), lithology (Calcareous, Clayey and Siliceous), region (Mediterranean and Atlantic), river (river name), coordinates (WGS84), and sampling date (from 8 May 2023 to 16 July 2024).
- *Physicochemical parameters*: temperature (°C), pH, dissolved oxygen (mg L^−1^ and % saturation), NPDOC (nitrogen, phosphorus and dissolved organic carbon), conductivity (µS cm^−1^), and nutrients [phosphate (mg PO₄³^−1^ L^−1^), nitrite (mg NO₂^−1^ L^−1^), nitrate (mg NO₃^−1^ L^−1^), ammonium (mg NH₄+ L^−1^), chloride (mg Cl^−1^ L^−1^), sulfate (mg SO₄²^−1^ L^−1^)].
- *Ecological quality indices*: Riparian Forest Quality Index (QBR), Specific Pollution Sensitivity Index (IPS) and Mediterranean reference criteria (MRC)^374–377^.
- *Other variables related to the pools*: shape (concave or cuboid), depth (m; mean and maximum), axes (m; major and minor axes of the pool and of the catchment area), degree of permanence (high, medium, low), permeability (high, medium, low), number of other pools within 200 m (count), and distance to the nearest pool (m). Permanence is the probability that the pool will re-form the following year. For example, it is more likely to be found again if the pool is located on bedrock than if it is located on pebbles. The permeability refers to the likelihood of water seeping into the sediment. For example, a pool is more likely to be watertight if it is built on bedrock than on sand.

## Technical Validation

DIATPOOL consists of 17 traits obtained both from external sources and from information generated specifically for this study.

On the one hand, 8 traits were obtained and harmonized from previously published literature. This includes Classical group^94^, Rafe group^89^, Morphology group^83^, Ecological guilds, If colonial, Solitary cells/Colonial type^14^, Teratological form^371^, as well as Ecological indicator values^27^. The use of these previously published traits ensures compliance with basic quality standards and provides valuable information for applications in disconnected pools. Furthermore, all these traits have been referenced by other studies, supporting their reliability as they have been previously tested and validated by members of the scientific community. Similarly, 4 traits were adapted for straightforward use and applicability in disconnected pools: Chloroplast number, Chloroplast shape, Dispersal capacity, and Habitat substrate. All of these traits were derived and subsequently adapted from the results presented by Nicolosi Gelis^87^.

On the other hand, 5 traits were specifically coded for this work: Size, Shape, Constriction, Habitat presence and Habitat tolerance. The design of these traits required the analysis of various diatom monographs, scientific articles and online resources. The different categories defined for Size, for instance, were derived from length and width measurements published both in original species descriptions and in other available references^14^. The coding of Shape and apical Constriction for each species required additional validation efforts by the authors. It was necessary to establish a consensus on the percentage of each trait category for all species. To address such variability, several diatom monographs and original species descriptions were reviewed^52,56,73^. Finally, the values for Habitat_presence and Habitat_tolerance were obtained after analyzing and comparing various previously published species lists from the main environments considered (aquatic, terrestrial, and aerial).

As mentioned in previously, the development of DIATPOOL required the incorporation of an initial taxonomic list of diatoms from disconnected pools. It was necessary to update and standardize numerous species names according to the most recent nomenclature (except for cases of taxonomic uncertainty) (AlgaeBase^96^). This included, for example, elevating some varieties to species rank (e.g., *Meridion circulare* var. *constrictum* to *Meridion constrictum*) and incorporating newly described species (e.g., *Sellaphora temporalis*). To ensure data comparability and transparency, DIATPOOL includes, at the end, part of the synonym considered during the compilation process. For all species, trait information was added whenever possible. It was necessary to add or update trait data both for species already included and for those absent from the original databases. This characterization was carried out using previously reviewed and published references, both for species and traits, which are recorded in the corresponding bibliographic columns of DIATPOOL. When the required references were not available, each species was carefully evaluated by experts to ensure accurate characterization. In cases where original or estimated information was provided (e.g., inferred from related species), it was explicitly indicated with notes such as “Present study” or “This information is considered an estimate based on expert judgment and the collective knowledge of the group.” All these efforts aimed to strengthen the transparency and credibility of the data. Nevertheless, important limitations remain in this study and should be carefully considered. First, we confirmed the existence of uneven information availability among taxa, which creates knowledge gaps and, in some cases, necessitates the use of expert-based annotations. Furthermore, the evolving nature of taxonomic uncertainty hinders a fully stable characterization of traits. These limitations should be understood within the context of each habitat, where optimal trait values may vary substantially, highlighting the need for further research tailored to specific environmental conditions. Therefore, DIATPOOL should be regarded as a dynamic resource, subject to continuous revision and the incorporation of new information.

The database of relative abundances (Abundance_diatom) and the habitat characterization database (Metadata_pool) are also made available to the scientific community. Both datasets have been thoroughly reviewed and optimized by the authors for application in various ecological analyses. Since our sampling was conducted in a standardized manner, the information provided by both matrices is ready to be used in a wide range of ecological studies.

**Fig. 3:**
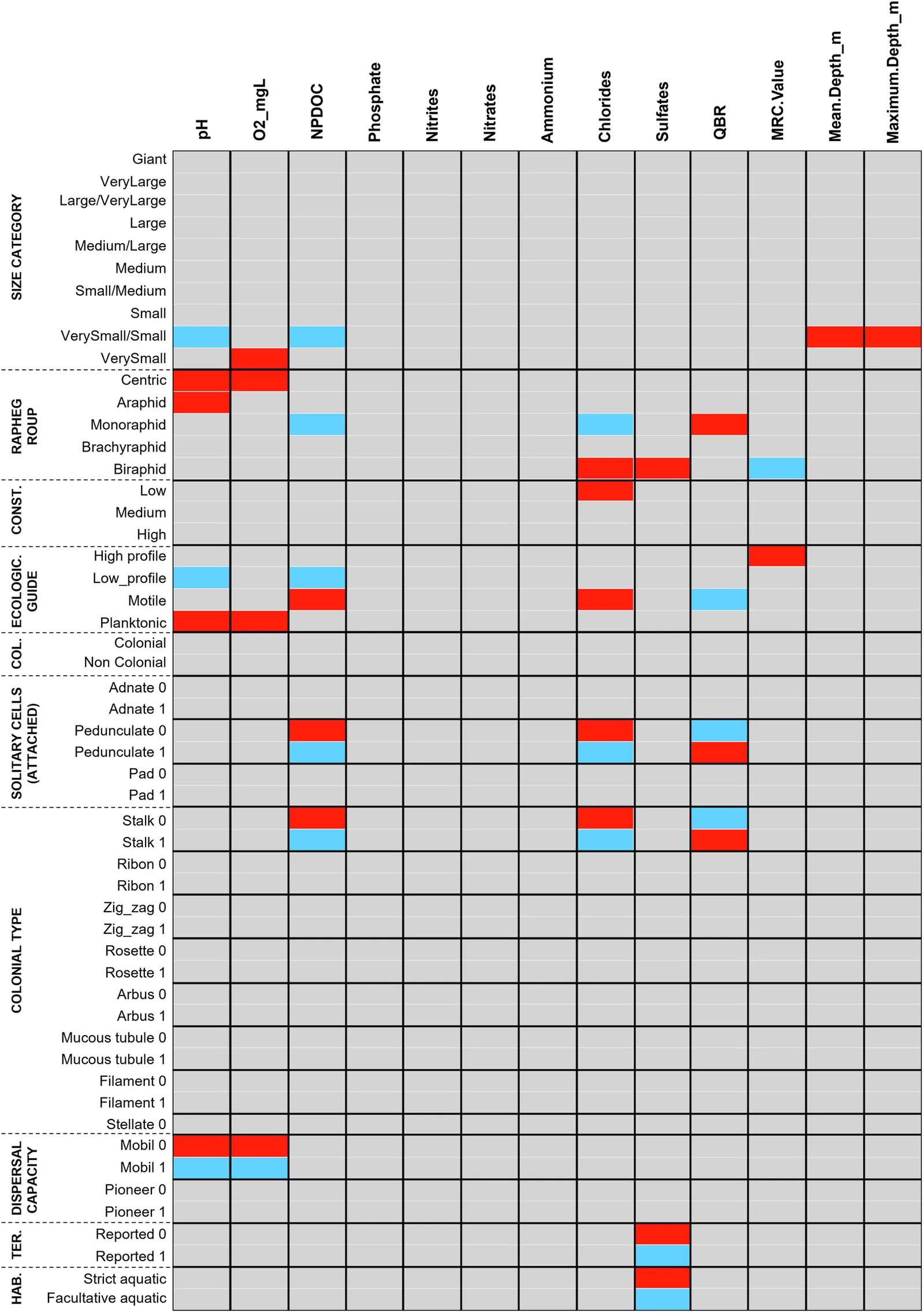
Results of the fourth-corner analysis showing the significant bivariate associations between environmental variables (Metadata_pool) and diatom traits (DIATPOOL database). Abundance data (Abundance_diatom) were log-transformed. Red cells represent significant positive relationships, blue cells indicate significant negative relationships, and non-significant relationships (gray cells).

To further validate DIATPOOL, a combined RLQ and fourth-corner analysis^40^ was carried out to relate diatom trait profiles in disconnected pools to environmental conditions. This analysis is a multivariate ordination technique capable of integrating three tables and detecting co-structures among a trait matrix (DIATPOOL), an environmental matrix (Metadata_pool), and a species matrix (Abundance_diatom)^378,379^. To facilitate data analysis and visualization, only a subset of traits and environmental variables were used (other combinations could reveal additional patterns related to diatoms in disconnected pools). Using 9999 random permutations and adjusted p-values, we identified significantly positive relationships (red cells), negative relationships (blue cells), and non-significant relationships (gray cells) between traits and environmental variables (Fig. 3). For example, in our size category, a positive relationship was observed between pool depth and very small/small diatoms, whereas pH and NPDOC showed a negative relationship. Conversely, both pH and oxygen concentration were positively associated with diatoms lacking a raphe (centric and araphid species). Additionally, higher chloride concentrations appeared positively related to strictly aquatic species, suggesting greater salinity tolerance in these taxa. These results highlight the value of DIATPOOL as a useful tool for ecological studies of diatoms in these habitats, enabling the analysis of distribution patterns and environmental adaptation.

## Data Availability

The DIATPOOL trait database (DIATPOOL.xlsx), the diatom relative abundance matrix (Abundance_diatom.xlsx), and the environmental metadata of the sampled pools (Metadata_pools.xlsx) are publicly available in a Zenodo repository at https://doi.org/10.5281/zenodo.20579411^106^. The repository contains all data files required to reproduce the analyses presented in this study.

## Code availability

No custom code was generated for this study. Statistical analyses were performed using standard functions implemented in R.
